# INTERNET USE AND NEGATIVE HEALTH PERCEPTIONS: THE MODERATING ROLES OF EDUCATION AND HEALTH LITERACY

**DOI:** 10.1093/geroni/igz038.1188

**Published:** 2019-11-08

**Authors:** Gul Seckin, Valarie Bell, Susan Hughes

**Affiliations:** University of North Texas, Denton, Texas, United States

## Abstract

There is a mixed support regarding the effect of Internet use on health and well-being. We estimated the extent to which e-health literacy predicted two domains of negative assessment of well-being: negative affect and self-reported experience of health problems. Respondents were randomly sampled from the largest national online probability-based research panel (N = 710). Hierarchical ordinary least squares regression analyses were employed for hypothesis testing. We computed interaction terms (e-health literacy x strain; e-health literacy x education; and education x strain) as determinants of negative subjective assessment of well-being. Older adults with higher levels of e-health literacy reported significantly more health information consumerism [(t (194) = 7.32, p ≤ .0001] but less strain in medical encounters [(t (194) = 2.92, p ≤ .004]. They reported less negative affect [(t (194) = 2.11, p ≤ .036] and more satisfaction with medical encounters [(t (194) = 4.70, p ≤ .0001]. The effect of perceived strain in medical encounters on negative affect was weaker among those with higher levels of education (β = -.314, p ≤ .01). Education had a significant moderating effect on the association between perceived strain in medical encounters and self-reported health problems, (β = -.550, p ≤ .05). Those who reported lower averages for e-health literacy but higher educational levels indicated lower averages on negative affect (β = -.597, p ≤ .05). Given that conventional methods of acquiring health-related information shift to the Internet, our study holds significant health and social implications for a rapidly growing Internet-connected older population.

